# Takayasu’s Disease With Bilateral Carotid Arteritis in a Caucasian Female: A Rare Presentation and Diagnostic Dilemma

**DOI:** 10.7759/cureus.19376

**Published:** 2021-11-08

**Authors:** Sanjay Yadava, Fatema Arafa, Zachary Shepherd

**Affiliations:** 1 Internal Medicine, State University of New York Upstate Medical University, Syracuse, USA

**Keywords:** vasculitis, hypertension, neck pain, carotid, takayasu's arteritis

## Abstract

Takayasu arteritis (TAK) is not an uncommon cause of vasculitis in Caucasian females, however, involvement of bilateral carotid artery is a very rare presentation. We are presenting a 31-year-old young Caucasian female who presented with left-sided neck pain, headache and was subsequently found to have vasculitis of bilateral carotid arteries.

## Introduction

Takayasu arteritis (TAK), also known as “pulseless disease,” is a large vessel vasculitis that primarily affects women of Asian descent, however, it is not uncommon in Caucasian females [1]. Clinical presentation is the result of ischemia secondary to larger blood vessel vasculitis, primarily affecting the aorta and its major branches. Symptoms are dependent on the vessels involved and include headache, dizziness, syncope, decreased pulses, and difference in systolic blood pressure between the two upper extremities [2]. Since most symptoms are nonspecific, TAK is often undiagnosed or misdiagnosed. In this case report, we highlight the difficulty in diagnosing Takayasu arteritis in a young Caucasian female who presents with neck pain.

## Case presentation

A 31-year-old female with a past medical history significant for preeclampsia and hypertension, who presented with left-sided neck pain and headache for about a month and worse for one week. She described her neck pain as throbbing, moderate to severe in intensity, worse with right-sided neck movement, and improved with lying on the left side of the neck. She also reported an episode of bilious vomiting prior to the hospital admission. She denied fever, chills, nausea, vomiting, neck stiffness, photophobia, blurring of vision, weakness or sensory loss, joint pain, photosensitivity, oral ulcer, family history of rheumatoid arthritis, systemic lupus erythematosus, or vasculitis. Physical examination was benign, except for mild tenderness to the left side of the neck with palpation, but no redness, swelling, lymphadenopathy, bruit, or decreased was pulsation noted.

**Figure 1:**
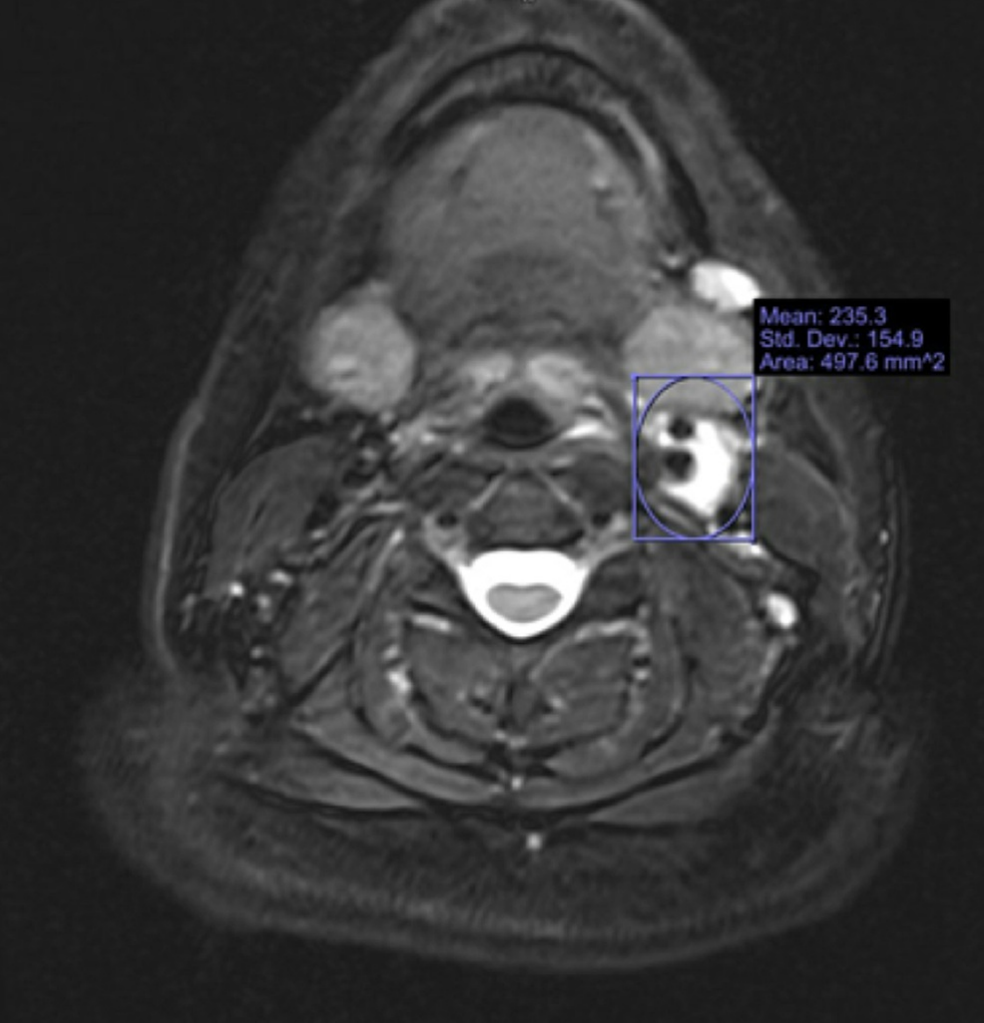
MRI soft tissue neck. Optical axial diffusion-weighted magnetic resonance image reveals hyperintensity and postcontrast enhancement surrounding the distal left common carotid, at the left carotid bifurcation, and proximal left internal carotid artery. Image obtained from Upstate Medical Department of Radiology.

Her lab work revealed normal complete blood count with white blood cell count of 7 × 10^3^/µL, hemoglobin/hematocrit of 14.1/41.8 g/dL, platelet count of 241; normal basic metabolic panel; and elevated sedimentation rate (ESR) to 24 (ref: <20 mm/hr) and C-reactive protein (CRP) to 11.8 (ref: <8 mg/L). Neurology was consulted and recommended CT angiogram (CTA) of head and neck that revealed no evidence of dissection or stenosis within bilateral carotids and no stenosis, aneurysm, or arteriovenous malformation in the anterior and posterior cerebral circulation. MRI neck soft tissue revealed hyperintensity and postcontrast enhancement and thickening of walls of the distal left common carotid at the left carotid bifurcation and proximal left internal carotid artery as well as mild thickening and enhancement of the wall of the right common carotid artery near the bifurcation, suggestive of vasculitis as shown in Figure 1. She was also evaluated by rheumatology. Rheumatological work-up was negative including antinuclear antibody (ANA), cyclic citrullinated peptide (CCP), centromere antibody, double-stranded DNA, histone, topoisomerase 1 (SCL-70), histidyl tRNA synthetase (JO-1), ribonucleoprotein (RNP), smith, Sjögren's-syndrome-related antigen A (SSA), and Sjögren's-syndrome-related antigen B (SSB) autoantibodies. Her immunoglobin levels (IgA, IgM, and IgG) and complement (C3, C4, and total) levels were also normal. Her infectious work-up including hepatitis B, hepatitis C, human immune deficiency virus, Epstein-Barr virus, COVID-19, syphilis serology, blood culture, and respiratory viral panel were also negative. She was started on a high dose of steroids along with trimethoprim-sulfamethoxazole prophylaxis as per rheumatology recommendations. She also had a CT scan of the thorax and abdomen with contrast to assess for vasculitis of other blood vessels, which were negative.

Her neck pain and headache improved slightly during the hospital stay and she was discharged home with a referral to an outpatient rheumatology clinic. There, the patient was diagnosed with TAK with carotidynia and secondary hypertension. The patient’s symptoms were well-controlled with 60 mg of prednisone daily. The patient subsequently transferred care to a local rheumatologist office and was placed on a prednisone taper. Patient was then started on Imuran 100 mg daily for TAK.

## Discussion

Takayasu arteritis is a chronic inflammatory disease of large to medium-sized vessels, which involves the aorta and its primary branches, pulmonary arteries, and coronary arteries. TAK is common in young Asian females [1]. TAK presents with a myriad of symptoms including weight loss, low-grade fever, fatigue, arthralgias, carotidynia, absent or weak pulses, hypertension, limb claudication, angina, rash, gastrointestinal, and neurological symptoms [2-6]. Lightheadedness, vertigo, syncope, orthostasis, headaches, convulsions, visual impairment, and strokes are common neurological symptoms with involvement of the carotid and vertebral arteries. Our patient presented with left-sided neck pain and tenderness and carotidynia, which is observed in 10-30 percent of patients at presentation [2]. She had been diagnosed with hypertension in the past, however, her blood pressure was fairly controlled with diet. CTA thorax/abdomen/pelvis was negative for aortic or renal artery involvement.

There is no single diagnostic test for TAK, usually the diagnosis is made on the consensus of clinical presentation, elevated inflammatory markers, and imaging studies suggestive of vasculitis involving large to medium-sized vessels. Biopsy of the blood vessel with immunohistochemistry is the most specific test, however, it is not pragmatic due to procedural risk. Elevated ESR and CRP support the diagnosis. Normal values of ESR or CRP should not deter a diagnosis of TAK. Imaging studies including magnetic resonance arteriogram (MRA) and CT angiogram (CTA) have a vital role in diagnosing TAK [7,8]. Vascular wall thickening and enhancement early in the disease, and arterial stenosis, occlusions, and aneurysms later in the disease involving medium to large-sized vessels are the common typical findings in imaging studies [9]. Our patient did have elevated ESR, CRP, and enhancement of the carotid artery which favor the diagnosis of TAK. According to American College of Rheumatology (ACR), patients need to have at least three of the six criteria as mentioned in Table 1. However, our patient has only two.

**Table 1:** Takayasu arteritis diagnosis criteria.

Need three or more of the following criteria:
Age at disease onset ≤40 years
Claudication of the extremities
Decreased pulsation of one or both brachial arteries
Difference of at least 10 mm Hg in systolic blood pressure between the arms
Bruit over one or both subclavian arteries or the abdominal aorta
Arteriographic narrowing or occlusion of the entire aorta, its primary branches, or large arteries in the proximal upper or lower extremities, not due to arteriosclerosis, fibromuscular dysplasia, or other causes

It is important to consider other likely diagnoses when considering TAK in this patient. Another potential diagnosis is fibromuscular dysplasia (FMD). Like TAK, the clinical presentation of FMD includes dizziness, hypertension, headaches, and even stroke [10]. FMD commonly involves the carotid and renal arteries [10,11]. As a result, it is often difficult to distinguish between TAK and FMD if an arterial biopsy is not available. However, unlike TAK, FMD is a noninflammatory disease, thus inflammatory markers like ESR and CRP are usually not elevated. Given that this patient had an elevated ESR and CRP and responded to steroids, it is more likely that her symptoms are a result of TAK as opposed to FMD [12].

Pathogenesis of TAK is poorly understood, however, cell-mediated chronic inflammation is thought to have a predominant role. Long-term immunosuppressive therapy is the main treatment. The immunosuppressive therapy includes glucocorticoids, which are often used in combination with other immunosuppressive agents, such as methotrexate, azathioprine, or mycophenolate mofetil. 

## Conclusions

Takayasu arteritis is not a common vasculitis in North America. Physicians should have a high index of suspicion for early diagnosis and institution of treatment.
